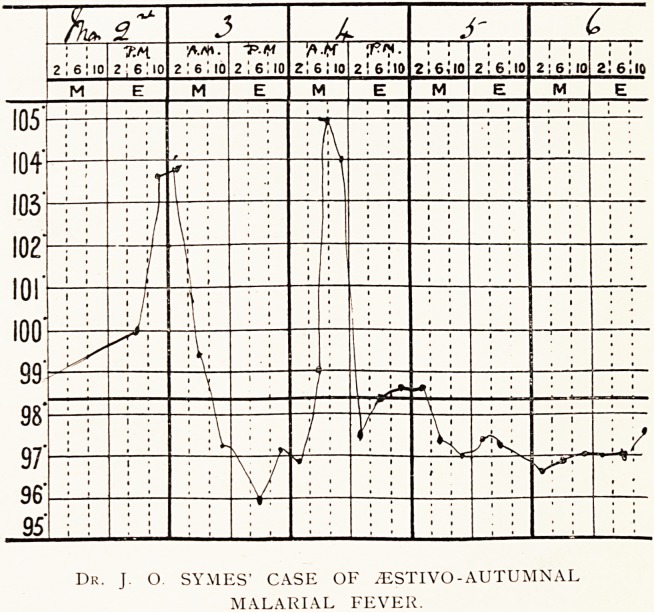# A Case of Æstivo-Autumnal Malarial Fever with Parasites of an Unusual Type

**Published:** 1904-06

**Authors:** J. Odery Symes

**Affiliations:** Assistant Physician and Bacteriologist, Bristol General Hospital


### A CASE OF ÆSTIVO-AUTUMNAL MALARIAL FEVER WITH PARASITES OF AN UNUSUAL TYPE.

BY

J. Odery Symes, M.D., D.P.H.,

*Assistant Physician and Bacteriologist, Bristol General Hospital.*

---

W. T., a seaman, aged 58, was admitted to the Bristol General Hospital on March 2nd, suffering from malaria. Some months previously he had arrived at Panama (Central America), and fearing fever, which was at the time peculiarly fatal there, he left his ship and tramped across the isthmus to Colon, where he worked for a few weeks. At Colon he developed fever, and was sent to hospital. In hospital he had daily attacks of ague, for which he was treated with quinine, "eight pills three times a day." The quinine caused deafness, but as the fever was not influenced he was shipped to Jamaica on his way to England. During the voyage he was still treated with quinine, but the fever became worse, and he was disembarked at Kingston and sent to the hospital. There was some slight improvement in his condition during his stay at Kingston, but he had daily attacks of ague and was finally shipped to Bristol. During his stay in Jamaica and on the homeward voyage he was regularly dosed with quinine. Throughout his illness he was troubled with dyspepsia and vomiting. On admission the patient was extremely weak, the mucous membranes were blanched, the skin of a bronzed yellow tint. There were a few hemorrhages in the legs. The spleen could be felt below the costal margin, and was tender. The heart was dilated. The patient could only retain liquid food, and this in small quantities. The tongue was thickly coated, the bowels constipated, and the abdomen distended. He complained of buzzing in the head, and could not hear the ticking of a watch placed one inch from the auricle. On the day following admission (March 3rd) the patient was given 5 grains of quinine and $\frac{1}{20}$ grain of arseniate of soda three times a day. He had an attack of ague in the morning, the temperature

Dr. J. O. SYMES' CASE OF ÆSTIVO-AUTUMNAL MALARIAL FEVER.

rising to 103.8°, and on the following morning during a second attack it rose to 105°. These were the only occasions on which fever was noted.

The blood was examined on March 4th. In the fresh blood numerous small intra-corpuscular non-pigmented parasites were seen, together with very many crescentic and ovoid bodies. The pigment in the crescents and ovoid bodies showed very lively movement, and this continued for more than an hour after the blood had been drawn. Flagellate bodies were observed to bud out from the ovoid bodies, and to move away in the serum. The peculiar feature of the blood was the presence of sausage-shaped parasites with scattered pigment lying within the red cells (fig. 4), and of cigar-shaped parasites lying across the corpuscle with their ends projecting beyond the periphery (fig. 3). Such forms of parasites have been described by Rowley[1]. Stained films showed the same forms, but the sausage-shaped bodies have not entirely preserved their original shape. Rowley thinks that possibly this elongated parasite is developed from ring-forms, and is destined ultimately to become a crescent. A differential blood count showed—Finely granular oxyphil leucocytes 55 per cent., coarsely granular oxyphil leucocytes 3 per cent., lymphocytes 37.5 per cent., myelocytes 2.5 per cent., nucleated reds 1 per cent.

The subsequent history of the case was uneventful. It having been determined that he was suffering from æstivo-autumnal fever, the administration of quinine was stopped (March 9th) and the arsenic alone administered. He was given a full diet, including milk, Mellin's food, custard, eggs, chops, etc. The deafness gradually disappeared, and he was sent to the Convalescent Home on March 19th. On the day of his discharge there were still a few crescentic and ovoid bodies in the blood. He states that there was a slight return of fever on March 26th, but this is uncertain. On March 28th no parasites could be found in the blood, and the patient appeared in excellent condition.

The case is primarily of interest on account of the unusual types of organism present, which differ from those I have seen in

[1] *Johns Hopkins Hosp. Bull.*, 1904, xv. 22.

other cases of æstivo-autumnal fever. It also illustrates the necessity of a microscopical examination of the blood in all cases of malaria, and the futility of treating cases of æstivo-autumnal malaria by quinine alone. The lymphocytosis is very characteristic but not absolutely diagnostic of malaria.

## Figures and Tables

**1 2 3 4 5 6 f1:**
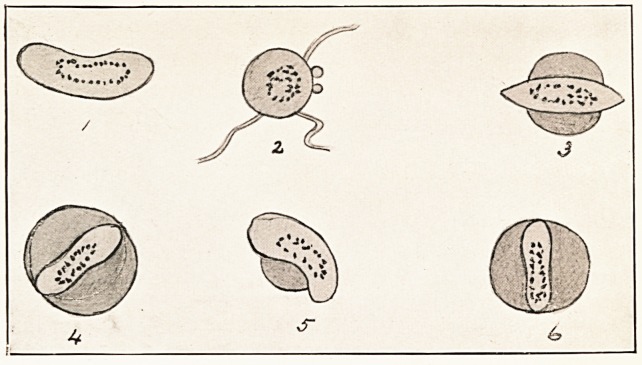


**Figure f2:**